# Development and psychometric assessment an instrument for investigating Women’s attitude toward home safety

**DOI:** 10.1186/s12889-022-13363-x

**Published:** 2022-05-12

**Authors:** Jalil Nazari, Rasoul Ahmadpour-geshlagi, Golam Reza Akbarinia, Neda Gillani, Fatemeh Karimkhani, Seyed Shamseddin Alizadeh, Jalil Nazari

**Affiliations:** 1 Department of Occupational Health Engineering, Health Faculty, Tabriz University of Medical Sciences, Golgasht St, Attar Neyshabori St, Tabriz, Iran; 2 Department of statistics and epidemiology, Faculty of health, Tabriz university of medical sciences, Tabriz, Iran

**Keywords:** Attitudes, Housewife, Home safety, Instrument

## Abstract

**Background:**

Approximately half of the Iranian population are women, and they play a vital role in the home. The women’s attitude can play a critical role in the safety of homes. Best of our knowledge, there is not a valid and reliable instrument to measure their attitude toward home safety. So, the present study aimed to design a psychometrics tool to assess women’s attitudes toward home safety.

**Methods:**

The researchers designed an instrument based on the home safety concept as the first instrument to measure housewives’ attitudes toward home safety. The developed instrument distributed among 686 women in Tabriz health centers. Content validity, confirmatory, and exploratory factor analysis were used to examine the construct validity, and Cronbach’s alpha and test-retest were employed to examine the reliability and reproducibility of the instrument.

**Results:**

In the face validity section, the impact score of all items was determined to be above 1.5. In the content validity section, 4 items were excluded from the 39 questionnaire items due to low Content Validity Ratio (CVR). The mean CVR of all items was 0.842. By conducting exploratory factor analysis, it was found that the questionnaire has six dimensions. Three questions were removed from the study due to lack of connection with other items. Also, Cronbach’s alpha coefficient of the questionnaire is equal to 0.924, which indicates the appropriate reliability of the instrument**.**

**Conclusions:**

This study aimed to develop a questionnaire to assess the safety attitudes of housewives toward home safety. It was found that the prepared tool has acceptable validity and reliability.

## Introduction

According to the World Health Organization (WHO), an accident is an event that occurs suddenly and unexpectedly, which leads to physical and mental injuries [1, 2]. Most of the accidents are preventable [2–4]. Home accidents occur within or around a home that can damage the house or resident. Falling, poisoning, electricity, choking, injuries, burns, and explosions are the most common accidents at home. These accidents could be due to different causes (e.g. poor home quality, children’s unsafe behaviors, and poor socioeconomic status) [1, 5].

Literature shows that approximately 25% of fatal accidents are home accidents [3]. Coty et al. (2015) examined the beliefs and practices of the elderly about fire safety in residential homes. They used ethnography and semi-structured interviews to investigate the beliefs of the elderly about fire safety [6]. Colver et al. investigated the impact of two health education approaches to improve the safety of children in residential homes using a randomized controlled trial [7]. Kendrick et al. (2013) examined the role of home safety training in controlling home accidents. Barbara et al. (2004) examined the factors that determine mothers’ safety performance at home in taking control measures to prevent accidental injury to children. They found that the factors influencing the control measures were more likely related to the type of injury [8].

People’s attitudes are crucial to home safety. In many Eastern cultures, such as Iran, women are responsible for household chores. For example, mothers play a significant role in preventing children’s accidents [9]. In general, women have two major roles (external and internal) in society. The external role includes activity in society, and the internal role is about motherhood and home [10]. As a result, women have a long presence in the home environment and are generally more responsible for housekeeping and taking care of their children, the elderly and domestic appliances. Therefore, it seems that women have a significant role in the safety of their homes. Consequently, investigating women’s attitudes toward home safety can help reduce these incidents. This study aimed to design and psychometrically evaluate the questionnaire on women’s attitudes toward safety at home.

## Materials and methods

### Subjects and study design

This study is a cross-sectional descriptive-analytic study. It was carried out from January to June 2019. The general population in this study is Iranian women, and the target population consists of all housewives in 2019. Also, the accessible population is Iranian housewives who have children and are referred to Tabriz city health centers to perform their own or their children’s health activities [11]. The sampling was multi-cluster type that in the first stage, 5 centers were randomly selected from 20 health centers in Tabriz. In the nex stage, Samples were randomly selected from within these clusters relative to the population of each cluster (in a health center with more housewives, more samples were selected). The questionary was pen-and-paper. Inclusion criteria were: having children, willing to participate in research, having at least a high school degree, and being over 18 years old. Exclusion criteria were evident mental illness (all target populations were previously registered with the health center, and the research team was able to identify people with mental health) and unwillingness to participate in the study. The purpose of the study and the importance of the study for home safety was explained to the participants participating in the study. The sample size needed to perform factor analysis to determine construct validity varies among researchers. Some literature considers a minimum of 200 for the sample size [12–15]. Klein also argues that exploratory factor analysis requires 10 or 20 samples per variable, but the minimum of 200 for sample size is also defensible [15]. Thirty-nine items were identified for the initial questionnaire using a literature review. Therefore, ten samples per tool item were selected (390 samples). The final sample size was 645, considering a 10% samples loss and a design effect coefficient of 1.5.

### Instrument

In the first step, terms or concepts equivalent to home safety was extracted through a literature review. Electronic databases, including Pro-Quest, Scopus, Google Scholar, Science direct, and SID and Magiran’s Persian-language databases from 2000 to 2019, searched for related studies. Studies in Persian and English were selected. The search string comprised of (home OR house) AND (checklist OR questionnaire OR instrument) AND (safety). Two experts collected the concepts from the literature. In total, 45 terms and expressions of the questionnaire were collected. Then, these concepts were merged and finalized by a third person. After reviewing, removing, and merging common phrases, 39 phrases remained. Finally, these items were turned into questions by the research team. All statements were designed on a 5-point Likert scale (strongly agree = 1, agree = 2, no idea = 3, disagree = 4, completely disagree = 5).

### Questionnaire validity

To determine the instrument’s validity, face validity, content validity, and construct validity of the made instrument were evaluated [16–18]. The designed questionnaire was presented to a panel of experts, including 9 Occupational Safety and Health PhD experts (8 of these experts were with a PhD and one of them was a PhD student, but all of them had at least 2 years of experience in this filed).

#### Face validity

Qualitative and quantitative methods were used to perform face validity. To determine qualitative face validity, ten experts were asked to comment on the level of difficulty, relevancy, and ambiguity of items in writing. Then, if the research team approved these comments, we would apply them to the item. For the quantitative approach, nine experts were asked to rate the importance of each item on a 5-point Likert scale: Completely important, important, moderately important, slightly important and not important. Then impact scores were calculated using formula 1, and items with scores less than 1.5 were excluded [19, 20]. Since all obtained impact scores were greater than 1.5 (mean score: 4.35), all items were retained for subsequent analysis.

1 $$\mathrm{Impact}\kern0.5em \mathrm{Score}\kern0.5em =\kern0.5em \mathrm{Frequency}\left(\%\right)\kern0.5em \times \kern0.5em \mathrm{Important}$$

In formula 1, frequency refers to the percentage of people who gave the item a score of 4 and 5, and importance is the average total score of people on importance based on the Likert scale.

#### Content validity

To determine content validity, both qualitative and quantitative methods were used based on the assessment of experts. The content validity ratio (formula 2) and content validity index (formula 3) were measured to assess quantitative content validity. In order to determine the content validity ratio, nine experts (different from prior step experts) were asked to examine each phrase on a three-part scale (necessary, useful but not necessary, not necessary). Due to the number of experts and considering the Lowsheh table, items whose content validity ratio was equal to or greater than 0.78 were retained [21, 22].

2 $$\mathrm{CVR}=\frac{\mathrm n{\displaystyle\frac{\mathrm N}2}}{\displaystyle\frac{\mathrm N}2}$$

In formula 2, “n” represents the number of experts who have chosen the necessary option and “N” represents the total number of experts. According to the content validity ratio decision table, if the panel of experts is nine people, the minimum acceptable validity for each item will be 0.78, which means that the relevant item with a significant level of reliability (*P <* 0.050) is essential in this tool [21, 23]. In the qualitative study of the questionnaire’s content, ten experts were asked to comment on issues such as Persian grammar, use of the right words, correct placement of items, and scoring in writing form. Then this comment was considered by the research team. In this regard, the questionnaire’s four items and rating system were corrected, and CVI and CVR were recalculated.

Waltz and Bausell method was used for Content validity [24]. For this purpose, a designed questionnaire was provided to the experts. They were asked to determine the relevance of each item in the questionnaire based on Waltz and Bausell content validity index. Thus, the degree of relevance, simplicity, and clarity was separately assessed for each item by experts using the 4-point Likert Scale. The content validity index was determined by formula 3. According to the Waltz and Bausell method, items with a score higher than 0.79 are appropriate, between 0.70–0.79 need revision, and less than 0.70 are unacceptable.

3 $$CVI\kern0.5em =\kern0.5em \frac{Number\kern0.5em of\kern0.5em reporters\kern0.5em who\kern0.5em have\kern0.5em selected\kern0.5em options\kern0.5em 3\kern0.5em and\kern0.5em 4}{Total\kern0.5em numbers\kern0.5em of\kern0.5em experts}$$

At this point, none of the items had scores below 0.7. Also, after revising, the CVI of one of the items ranged from 0.70 to 0.79. Consequently, the content validity index of the item was re-evaluated for the second time by experts. The mean content validity index of the questionnaire was 0.94 and is considered appropriate based on the opinion of Polit and Beck [25].

#### Construct validity

The two-stage strategy of Muliak and Millsap model was used to determine the construct validity [26]. For doing Exploratory Factor Analysis (EFA) and Confirmatory Factor Analysis (CFA) a split-half method was used. In the first step, exploratory factor analysis was used to extract factors (latent variables). Kaiser-Meyer-Olkin (KMO) was performed to check the adequacy of the samples. The Bartlett Test of Sphericity was used in the sample to ensure that the correlation matrix underlying the factor analysis is not zero. Values ​​above 0.7 in the KMO test and *p*-value less than 0.050 in Bartlett’s test were considered as the criterion of suitability for factor analysis [27]. The principal component method with direct oblimin was used for data exploratory factor analysis. To determine the number of main factors of the questionnaire, three indices, including eigenvalue, fine-grained diagram, and contribution of each factor to the sum of the total variance, were used. The milestone of 0.3 was considered the minimum factor load needed to maintain each expression in the factors extracted from factor analysis [28]. After extracting the factors and expressions in each factor, the degree of consistency of these factors with the main concept and dimensions was investigated. As a result, two sentences were omitted, and the instrument’s expression count reached 37. In the second step, confirmatory factor analysis was used to evaluate the relationships between the indicator and latent variables to validate the EFA model on a sample separately from the exploratory step. Confirmatory factor analysis shows whether tool items are appropriated and fitted to relevant factors based on theoretical expectations. The estimated method was maximum likelihood [26].

### Confirmatory factor analysis

Structural equation modelling with confirmatory factor analysis was used to test the relationships between variables and the instrument’s psychometric properties. Model fit was evaluated using the chi-square statistic (χ2), chi-square ratio, and degrees of freedom (χ2 / df). Goodness-of-fit index (GFI), adjusted goodness-of-fit index (AGFI), mean square root approximate Error (RMSEA) and CFI > 0.9, χ2 / d < 5, GFI > 0.9, and AGFI≥0.8, RMSEA < 0.08 are considered as appropriate indices and reasonable values [26].

### Questionnaire reliability

**Fig. 1:**
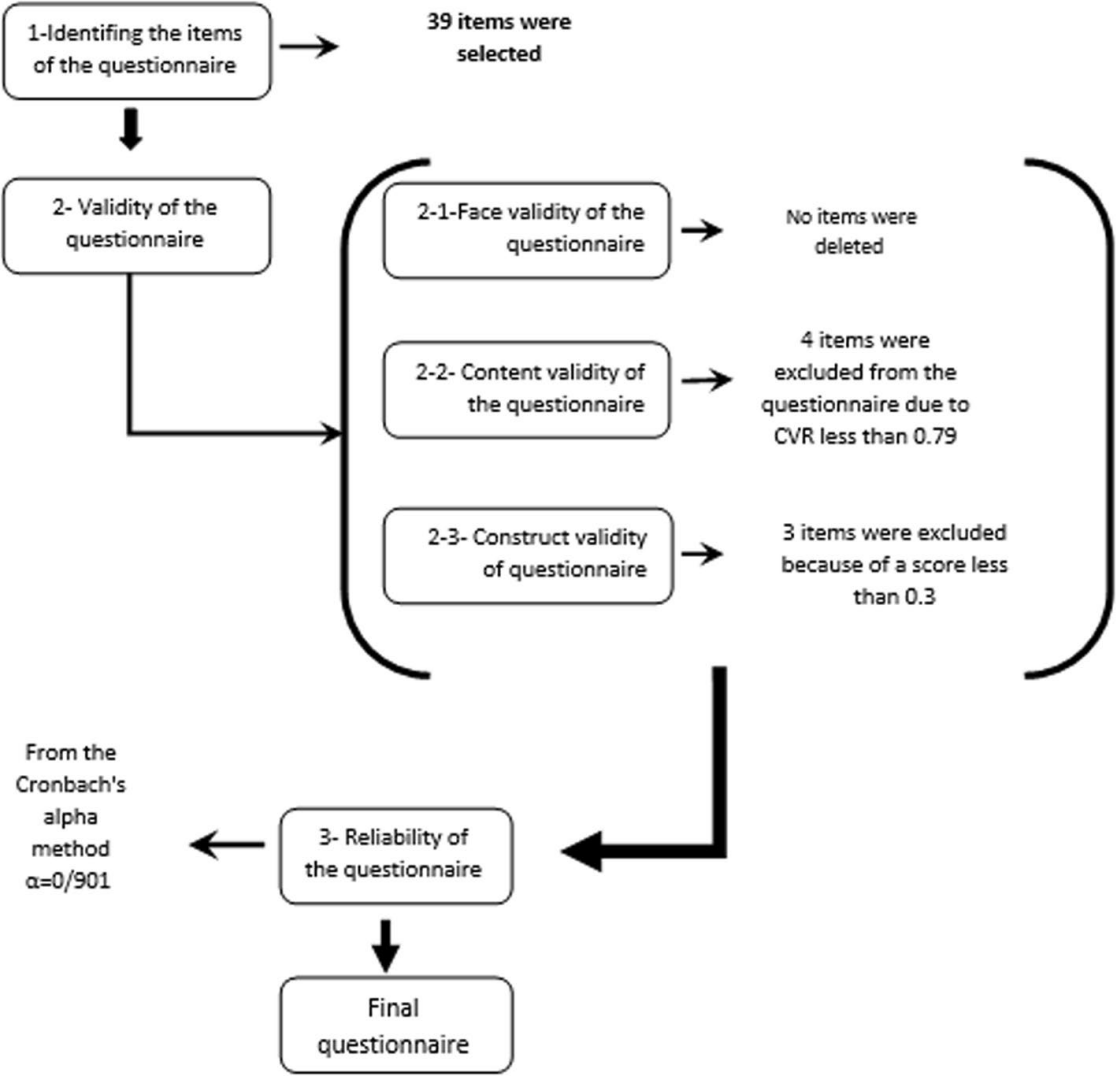
Study Method (N represents the number of items in each step)

The reliability of the questionnaire means to what extent the questionnaire yields the same results under the same conditions [29]. The reliability of the study questionnaire was calculated using Cronbach’s alpha coefficient and test-retest. Therefore, 20 participants were asked to complete the questionnaire in two steps with 2 weeks intervals [30]. Cronbach’s alpha coefficient was used to investigate the internal consistency of the questionnaire. An alpha coefficient greater than or equal to 0.70 was considered as a satisfactory criterion [31]. The reliability of stability and test-retest of the questionnaire were also assessed by repeated sampling and by calculating the intra-class correlation coefficient (ICC) [32]. The whole process of designing and developing the questionnaire is shown in Fig. 1.

## Results

### Results of statistical analysis

The mean age of participants in the panel of experts was 37.22 (4.96), and the mean of their work experience was 10.66 (4.55) years. One of the experts had a master’s degree, and the rest had a PhD. Of 800 distributed questionnaires for the construct validity survey, 686 (86% of the participants) were returned. The mean age of participants was 37.56 (7.53) years. Other demographic characteristics of the study population are listed in Table 1.

**Table 1:** Demographic characteristics of the study participants

Individual Social Variables (Quantitative phase of the study)	Subgroup	Frequency (Percent)
Marital status	Married	637 (92.9)
	Single (Deceased spouse or divorced)	18 (2.6)
	It was not announced by the participant	31 (4.5)
Education	High school	106 (15.5)
	Diploma	285 (41.5)
	Associate degree	57 (8.3)
	Bachelor	143 (20.8)
	MSc	38 (5.5)
	Above MSc	6 (0.9)
	It was not announced by the participant	51 (7.5)
Employment status	Employed	257 (37.5)
	Housewife	378 (55)
	It was not announced by the participant	51 (7.5)

Collected data were analyzed using IBM SPSS Statistics for Windows, Version 24.0. (Armonk, NY: IBM Corp; 2016). The variables were described as Mean (SD) and frequency (percent). The skewness indices (absolute values less than 3) and kurtosis (absolute values less than 10) along whit Q-Q plot, were considered to evaluate the normality of the quantitative variables [26, 28]. A *p*-value of less than 0.050 was considered statistically significant in all tests. Finally, a total of 39 items were selected by the literature review. Four questions were excluded in the content validity section due to a CVR of less than 0.78. The mean CVR of all items was 0.842.

### Confirmatory factor analysis results

In the construct validity section, the KMO sampling index value (0.935) is at the optimum level [33], and also the Bartlett, The test of Sphericity with a score of 6414.941 is significant (*p* ≤ 0.000) [34]. Therefore, it is justified to perform factor analysis. Through factor analysis, seven dimensions were extracted, two of these seven dimensions having the same concepts and were placed in the same dimension (child safety). Three questions were excluded from the study for lack of relevance to any of the dimensions. Thus, the final questionnaire consisted of 32 questions and six dimensions as follows (Table 2):

- Prevention dimension (F3): 5 questions
- Safe thinking dimension (F1): 7 questions
- Child safety dimension (F4): 6 questions
- Commuting safety dimension (F5): 5 questions
- Home safety requirements dimension (F2): 5 questions
- Hazard identification dimension (F6): 4 questions

**Table 2:** Questionnaire content validity index (CVR and CVI)

Dimension	Question	CVR	CVI
**Prevention**		**0.82**	**0.90**
	Access to water and gas valves is essential for everyone in emergency cases.	0.78	0.89
	I need to know the emergency numbers.	1	0.89
	When buying and consuming food, it should pay attention to the date of its consumption.	0.78	0.82
	Occasionally, the leakage of gas valves should be monitored.	0.78	0.93
	Knowing the fire station contact number is essential for anyone.	0.78	1
**Safe thinking**		**0.93**	**0.95**
	Occasionally, the power cord cladding must be checked to be safe.	0.78	1
	Winning and hazardous corners of furniture must be covered with veneer.	1	1
	Improper lifting and handling of equipment can be harmful.	1	0.96
	Bathroom and toilet ventilation should be inspected occasionally.	1	0.89
	When locating home appliances, I notice the likelihood of people getting stuck and falling.	1	1
	It is important to check for the possibility of slippery floors before entering the bathroom and toilet.	0.78	0.93
	Occasionally, it is important to check that wall and ceiling fixtures (photo frames, boards, chandeliers, etc.) are secure.	1	0.89
**Child safety**		**0.89**	**0.97**
	The baby bed must have a protective fence.	1	1
	It is important to keep detergents out of the reach of children.	1	1
	Keeping the baby alone is dangerous in the bathroom and toilet.	0.78	0.96
	It is important to keep the lighters out of the reach of children.	0.78	0.96
	Kitchen is not a good place for kids to play.	0.78	0.93
	Children are not allowed to use sharp and winning instruments.	1	1
**Commuting safety**		**1**	**1**
	Lighting of corridors is important when commuting.	1	1
	It is important that there are no obstacles to commute in the corridors.	1	1
	The stairs must have a protective fence.	1	1
	The elevator needs to be periodically checked.	1	1
	It must be assured that the elevator warning keys are working correctly.	1	1
**Home safety requirements**		**0.95**	**0.91**
	A fire extinguisher is essential for home.	1	0.95
	A first aid kit is essential for a home.	1	1
	Emergency exit routes are essential for a residential building.	0.78	0.85
	Awareness of safe places in the home is essential for sheltering when an earthquake occurs.	1	0.84
	At night, light in a small amount is needed to commute indoors.	1	0.93
**Hazard identification**		**0.89**	**0.93**
	In bathrooms and toilets, there is a risk of electric shock.	1	0.93
	The height of the balcony is effective in falling.	0.78	0.89
	There is a possibility of slipping on the kitchen floor.	0.78	0.96
	The layout of kitchen appliances plays a role in the occurrence of accidents.	1	0.96

### Conceptual model

After performing the factor analysis process using IBM SPSS Amos 24.0 software, the model fit was evaluated according to the model output. Based on factor analysis, the fitting of the final model confirmed based on the following indices [27, 28]:

RMSEA = 0.048 < 0.08 and; CFI = 0.91 > 0.9; GFI = 0.91 > 0.9.

AGFI = 0.88 > 90 and IFI = 0.89 < 1, x^2^/df = 2.53 < 5.

**Fig. 2:**
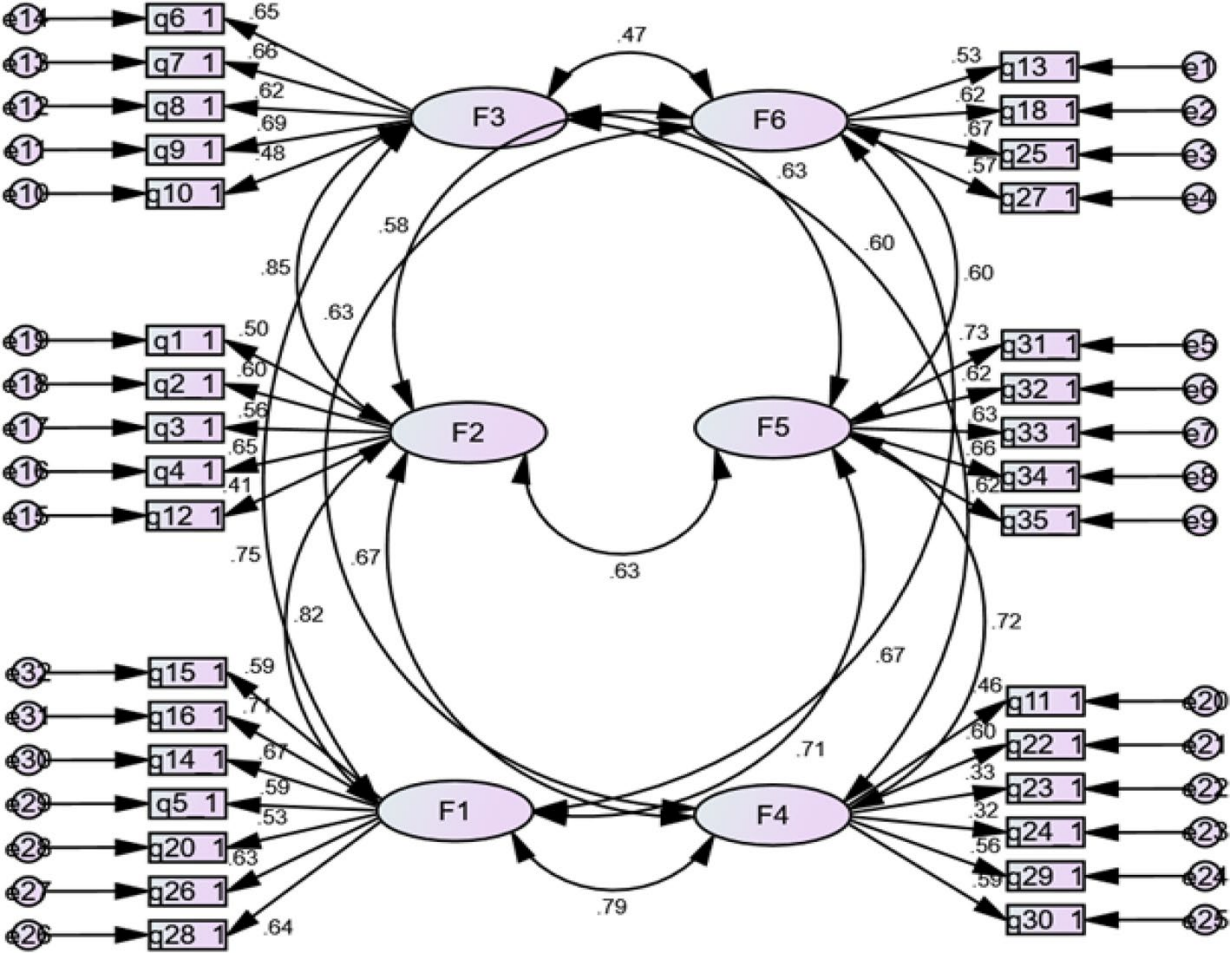
Relations between items and factors and between factors (based on confirmatory factor analysis). All relations between factors and items as well as between the factors were significant (*P* < 0.050)

Evaluation of the relationship between parameters and factors based on the final model showed that the items have significant loading on six factors. As shown in Fig. 2, the standardized factor loadings range from 0.32 to 0.85. According to the figure, all variables have a high correlation with their respective constructs. Question 23 (0.32) variables had lower correlations with other factor 5 variables and question 10 (0.48) variables than other factor 3 variables (prevention).

### Reliability outcome

Cronbach’s alpha coefficient of the whole instrument was 0.901. The Cronbach’s alpha coefficient of each dimension is presented in Table 3. Three dimensions of the questionnaire have acceptable reliability, but the other three have low reliability, so the tool has relative internal reliability. The Intraclass Correlation Coefficient (ICC) ranged from 0.993 to 0.996 for all variables, confirming test-retest reliability. The results of the structural model evaluation and final questions are presented in Table 3. The final questionnaire consisted of 32 questions. The questionnaire items classified on a 5-point Likert scale from strongly agree to strongly disagree. Because the questionnaire was a 5-point scale, the options were scored from 1 to 5 (160–32). By adding the scores of each question, the scale score was obtained. Finally, by summing the scores of all scales, the individual’s attitude score was calculated.

**Table 3:** Factor analysis result, Cronbach’s alpha coefficient and final questionnaire questions

Dimension	Question	Score	Cronbach’s alpha
**Prevention**			0.738
	Access to water and gas valves is essential for everyone in emergency cases.	−0.617	
	I need to know the emergency numbers.	−0.587	
	When buying and consuming food, it should pay attention to the date of its consumption.	−0.565	
	Occasionally, the leakage of gas valves should be monitored.	−0.484	
	Knowing the fire station contact number is essential for anyone.	−0.508	
**Safe thinking**			0.815
	Occasionally, the power cord cladding must be checked to be safe.	0.570	
	Winning and hazardous corners of furniture must be covered with veneer.	0.728	
	Improper lifting and handling of equipment can be harmful.	0.675	
	Bathroom and toilet ventilation should be inspected occasionally.	0.352	
	When locating home appliances, I notice the likelihood of people getting stuck and falling.	0.417	
	It is important to check for the possibility of slippery floors before entering the bathroom and toilet.	0.318	
	Occasionally, it is important to check that wall and ceiling fixtures (photo frames, boards, chandeliers, etc.) are secure.	0.313	
**Child safety**			0.624
	The baby bed must have a protective fence.	−0.473	
	It is important to keep detergents out of the reach of children.	−0.780	
	Keeping the baby alone is dangerous in the bathroom and toilet.	−0.583	
	It is important to keep the lighters out of the reach of children.	−0.474	
	Kitchen is not a good place for kids to play.	−0.788	
	Children are not allowed to use sharp and winning instruments.	0.808	
**Commuting safety**			0.804
	Lighting of corridors is important when commuting.	−0.660	
	It is important that there are no obstacles to commute in the corridors.	−0.645	
	The stairs must have a protective fence.	−0.666	
	The elevator needs to be periodically checked.	−0.817	
	It must be assured that the elevator warning keys are working correctly.	−0.697	
**Home safety requirements**			0.648
	A fire extinguisher is essential for home.	−0.717	
	A first aid kit is essential for a home.	−0.611	
	Emergency exit routes are essential for a residential building.	−0.559	
	Awareness of safe places in the home is essential for sheltering when an earthquake occurs.	−0.617	
	At night, light in a small amount is needed to commute indoors.	−0.302	
**Hazard identification**			0.697
	In bathrooms and toilets, there is a risk of electric shock.	0.758	
	The height of the balcony is effective in falling.	0.523	
	There is a possibility of slipping on the kitchen floor.	0.822	
	The layout of kitchen appliances plays a role in the occurrence of accidents.	0.523	

## Discussion

Women in Iran and similar countries have a decisive role in the arrangement, decoration and protection of the home. Therefore, it is very important to examine their attitude towards home safety. This will be very helpful in determining remedial action (e.g. training). Previous studies have focused mostly on accident statistics and their types (1, 4, 5). Best of our Knowledge, no instrument has been introduced to examine people’s attitudes toward basic safety principles at home. Therefore, this study aimed to design and psychometrically evaluate a questionnaire to determine women’s attitudes toward basic safety principles at home. The CVI was 0.84 and the CVR was 0.94, which is quite appropriate considering the number of expert panels (*n* = 9). The reported results for CVI and CVR are appropriate (22–24). Also, the Adaptive Fit Index (CFI) results indicate the suitability of the model [28]. The factor loadings of the questions posed for each structural factor in confirmatory factor analysis were above 0.5. This finding indicates that factors are well connected to questions [35]. Also, the values obtained for RMSEA, GFI, and x^2^/df were 0.048, 0.91, and 2.53, respectively, all of which values, together with CFI, could indicate the suitability of the used model [26, 28].

Cronbach’s alpha criterion was used to assess the reliability of the questionnaire. Cronbach’s alpha coefficient was 0.901, which is higher than the acceptable value of this index (0.7). This value indicates that the internal correlations between the questionnaire questions are high, and therefore the questions asked are homogeneous [36]. Also, the intra-class correlation coefficient results show that the designed instrument can have similar results at different times [37]. Therefore, the final questionnaire has acceptable validity and reliability.

When developing questionnaires, there was some error (e.g. coverage error, sampling error, non-response bias, measurement error) [38, 39]. Researchers are aware of these errors and try to reduce them as much as possible. Our respondent rate was 86%, while Babbie (1998) suggests that a response rate of 70% or more is perfect [40]. Measurement errors in the survey may be caused by interviewers, respondents, data processors, and other survey personnel [39]. The researcher tried to use a component team to lower the effect of this error. The respondents may feel threatened by controversial questionnaire items or their sensitive nature, such as race, gender, or income. So, the study was explained to all participants, and the anonymous questionary was used; the principle of confidentiality was observed, and the research team tried to use positive or neutral questions [40, 41].

## Conclusion

This study aimed to develop a questionnaire to assess the safety attitudes of housewives toward home safety. It was found that the prepared tool has acceptable validity and reliability. In this regard, governments and private institutions can benefit from the results of this questionnaire for planning to reduce housing accidents.

### Limitations and future studies

The questionnaire was pen-and-paper, and all participants had at least a high school degree. As the accessible population were registered in health centers, the researcher used purposive site selection, which could lead to both coverage and sampling error [42]. Researchers can use this questionnaire in future studies to examine women’s attitudes toward home safety in different societies and to examine possible factors affecting this attitude, including education level and household income. Also, by examining the validity and reliability of this questionnaire in another group of society, a tool can be obtained to examine the attitudes of different groups in society towards home safety.

## Data Availability

All data used for the analysis are available from the corresponding author upon request.

## Abbreviations

KMO: Fat Kaiser-Meyer-Olkin
CVI: Content Validity Index
CVR: Content Validity Ratio
WHO: World Health Organization
