# Correction: Development and psychometric assessment an instrument for investigating Women’s attitude toward home safety

**DOI:** 10.1186/s12889-022-13446-9

**Published:** 2022-07-11

**Authors:** Rasoul Ahmadpour-geshlagi, Golam Reza Akbarinia, Neda Gillani, Fatemeh Karimkhani, Seyed Shamseddin Alizadeh, Jalil Nazari

**Affiliations:** 1 Department of Occupational Health Engineering, Health Faculty, Tabriz University of Medical Sciences, Golgasht St, Attar Neyshabori St, Tabriz, Iran; 2 Department of statistics and epidemiology, Faculty of health, Tabriz university of medical sciences, Tabriz, Iran

**Correction: BMC Public Health 22, 948 (2022)**

**https://doi.org/10.1186/s12889-022-13363-x**

The author ‘Jalil Nazari’ was published twice on the original publication of this article. The article [1] has been updated to amend this error.
